# Risk factors for newly-developed cardiovascular disease and quality of life during the COVID − 19 pandemic: an analysis of the English longitudinal study of ageing

**DOI:** 10.1186/s12889-023-16135-3

**Published:** 2023-07-05

**Authors:** Mubarak Patel, Olalekan Uthman

**Affiliations:** 1 Warwick Evidence, Warwick Medical School (WMS), University of Warwick, Coventry, CV47AL UK; 2 Warwick Medical School (WMS), University of Warwick, Coventry, CV47AL UK

**Keywords:** Heart disease, Risk prediction, Wellbeing, Pandemic, Lockdown

## Abstract

**Introduction:**

The COVID-19 pandemic had a wide range of effects on the English population, including on health and quality of life due to the subsequent lockdown restrictions set.

**Aims:**

To investigate longitudinal changes in developing cardiovascular disease (CVD) and how that affects quality of life from pre-pandemic and during two lockdowns in England, in adults aged 50 years and above, and what factors are associated with this.

**Methods:**

Wave 9 of the core English Longitudinal Study of Ageing (ELSA) and Waves 1 and 2 of the ELSA COVID-19 sub-study were used to investigate the factors associated with developing CVD between timepoints, and what factors alongside CVD are associated with quality of life.

**Results:**

Higher age and depression were associated with newly-developed CVD from pre-COVID to both COVID sub-study waves. Additionally, body mass index (BMI) increased odds of CVD and physical activity decreased odds. Non-White ethnicity, depression, females, and developing CVD were lower associated with quality of life. Decreased age and increased physical activity were associated with higher quality of life.

**Discussion:**

Ethnicity was not associated with newly-developed CVD but was associated with quality of life. Other factors of importance include age, depression, gender, and physical activity. Findings are informative for future risk stratification and treatment strategies, especially while the COVID-19 pandemic is ongoing.

## Introduction

The coronavirus disease 2019 (COVID-19) pandemic is an ongoing worldwide pandemic caused by the severe acute respiratory syndrome coronavirus 2 (SARS-CoV-2), and is responsible for the deaths of over 6.6 million people out of 650 million confirmed cases [1] globally. In the UK alone, it is responsible for 213,000 deaths out of 24 million cases. Common symptoms of COVID-19 include fever, coughs, fatigue, and a new loss of taste or smell, which generally subside in one or two weeks.

In a number of patients, however, COVID-19 has effects well after the period of infection has passed. ‘Long COVID’ refers to the presence of symptoms far longer than it would be expected after a patient has recovered from the SARS-CoV-2 infection [2]. It can affect multiple organs and systems such as the respiratory, neurological, gastrointestinal, musculoskeletal, and cardiovascular systems [3]. For example, older individuals with long COVID have significant pulmonary impairment [4]. This is particularly important in an ageing population since they are already at higher risk of conditions such as cardiovascular disease (CVD) due to their advancing age [5], lower levels of physical activity [6], and other comorbidities [7], among other factors [8, 9].

A meta-analysis of over 18 million total patients [10] found that individuals of Black and Asian ethnicity are at an increased risk of COVID-19 infection compared to White individuals, with Asians at a higher risk of intensive care and death. Conversely, a different systematic review of almost 18 million patients did not conclude that membership of a particular ethnic minority group as an independent poor prognostic factor for COVID-19 [11]. Furthermore, studies have attempted to investigate the link between COVID-19, ethnicity, and poor outcomes such as mortality and hospitalisation. One study found that Asians and Pacific Islanders had slightly lower odds of a major adverse cardiovascular event compared to Non-Hispanic Whites [12]. Another [13] found that those of South Asian ethnicity appear at risk of worse COVID-19 outcomes compared to Whites in the UK, including hospitalisation and death, whereas another found no different between South Asian and White with respect to COVID-19-related mortality [14].

Cardiovascular diseases (CVD) are a group of disorders of the heart and blood vessels. They are the leading cause of death worldwide [15], a major cause of disability [16], and are associated with a reduction in quality of life (QOL) [17, 18]. A German study [19] found QOL in CVD patients were generally comparable with the general German population, however there was a negative correlation of QOL with age, suggesting QOL is older CVD patients deteriorates faster than in the general population. Moreover, the presence of other comorbidities, such as diabetes, alongside CVD was significantly associated with a lower QOL [20].

A systematic review of 12 studies [21] found that the impact of COVID-19 on health-related quality of life (HRQOL) was substantial, with large decreases in many QOL domains from before to after the COVID-19 lockdown period started. Furthermore, this review found significant changes in HRQOL were most common in women and those aged 60 years or more. Another study of over 1000 UK respondents found significant increases in mental health issues such as depression and anxiety in the UK relative to pre-pandemic [22]. Further studies have arrived at a similar conclusion where the COVID-19 outbreak and the subsequent stay-at-home orders have taken tolls on people’s mental health and QOL [23–25].

The emergence of the COVID-19 pandemic brought significant disruptions in daily routines, economic stability, and healthcare services, ultimately leading to heightened stress levels and indirect health risks. This research aims to explore the potential links between the COVID-19 pandemic and the risk factors for newly developed cardiovascular disease (CVD) while considering its impact on the quality of life in the aging population, specifically referencing the English Longitudinal Study of Ageing.

Numerous studies have highlighted the negative effects of the pandemic on overall mental health, with increased stress, depression, and anxiety levels reported across various demographic groups [26]. Moreover, such psychological distress can contribute to increased risk factors for cardiovascular diseases such as high blood pressure, smoking, unhealthy diet, and physical inactivity [27]. Given the link between chronic stress, psychological wellbeing, and cardiovascular health, it is crucial to explore the specific impact of the COVID-19 pandemic on CVD risks.

COVID-19 has also presented unique challenges for the elderly population who have experienced isolation and decreased physical activity due to social distancing measures [28]. This is particularly pertinent given that the aging population is already predisposed to cardiovascular disease, primarily due to age-related physiological changes and increased prevalence of risk factors such as hypertension and diabetes [29]. Therefore, understanding the influence of COVID-19 on CVD risks among the aging population is crucial in informing public health interventions and policy.

Finally, the Quality of Life (QoL) is an essential health outcome that reflects a person’s perception of their position in life. It encompasses their physical health, psychological state, personal beliefs, and relationships [30]. The pandemic has widely disrupted QoL due to the restrictions on daily activities, travel, and social relationships [31]. Given that cardiovascular health and QoL are intrinsically linked [32], it is essential to investigate the interplay between these factors during the pandemic.

This study aims to bridge this gap in knowledge by providing a comprehensive analysis of the risk factors for newly developed cardiovascular disease and the quality of life during the COVID-19 pandemic, focusing on the aging population in England. Therefore, the aims of this study were to (a) explore which factors increased the risk of newly-developed CVD and (b) explore which factors affect QoL during the COVID-19 pandemic, comparing data from two to three years prior to the pandemic and two separate timepoints during the first two lockdowns in England.

To present, no studies have examined the factors related to developing CVD for the first time during the COVID-19 lockdown in the UK and how that, adjusting for important confounding variables, is related to changes in quality of life before and after the start of the pandemic. This study aimed to be one of the first which investigated how QOL changed as a result of a CVD diagnosis in adults aged 50 years or over which will allow the identification of the most at-risk groups, especially during a time of uncertainty and strain for health services which was the COVID-19 pandemic.

## Methods

### Data sources

Data used in this paper were drawn from the English Longitudinal Study of Ageing (ELSA) project, which collects representative data from people aged 50 years and over to understand various aspects of ageing in England [33]. The sample has been refreshed over several waves so that it remains representative of the English over 50 population. The relevant wave from the main ELSA study for the following analysis is Wave 9, which was collected between 2018 and 2019. As part of an effort to understand the effects of the COVID-19 pandemic on the older population in England, the ELSA COVID-19 sub-study was conducted during June-July 2020 (COVID wave 1) and November-December 2020 (COVID wave 2). All ELSA data is publicly available at: https://beta.ukdataservice.ac.uk/datacatalogue/studies/study?id=8688#!/.

### Outcomes

Participants in the COVID-19 sub-study were asked the following at both waves: “Thinking about what has happened since we last saw you (i.e. ELSA Wave 9 for relevant participants), has a doctor ever told you that you developed a new health condition?” Relevant responses for this paper included hypertension, angina or heart attack, congestive heart failure, or stroke. These were combined to form a binary variable of newly-developed CVD.

Subjective quality of life in later life was evaluated using two different versions of the Control, Autonomy, Self-realisation, and Pleasure scale. The 12 item (CASP-12) questionnaire is a shorter version of the CASP-19 and excludes questions 3, 6, 8, 13, 14, 16, 17 from the larger CASP-19 questionnaire. The 12-item one was used in the two Covid waves whereas the original CASP-19 questionnaire was used in the original waves of ELSA. Responses were based on a 4-point Likert scale: 0 = never, 1 = not often, 2 = sometimes, and 3 = often. Negatively perceived questions are reverse scored. All responses are summed, yielding a score with range 0 to 36, where higher scores indicate high levels of satisfaction of quality of life. The 12-item scale has not been validated against the 19-item scale. However, to allow comparison between ELSA Wave 9 and the COVID-19 sub-study waves, raw data from Wave 9 was converted from CASP-19 to CASP-12 by using the 12 questions that appear in CASP-12 to calculate the QoL score at Wave 9. To test how appropriate this was, the correlation between CASP-12 and CASP-19 scores were calculated, as well as Cronbach’s alpha to measure internal consistency of the 12-item scale.

### Exposure variables

Existing CVD, defined as CVD at Wave 9, was based on whether or not participants had answered questions regarding having experienced angina, myocardial infarction, congestive heart failure, or stroke by ELSA Wave 9. Newly-developed CVD was based on participants who did not have CVD at Wave 9 but developed any of the aforementioned CVD conditions during the two COVID-19 sub-study waves.

Ethnicity in ELSA was reported as either ‘White’ or ‘Non-white’. Specific non-white ethnicity was grouped to protect the identities of participants. Ethnicity was one of a number of variables that were fed-forward from ELSA Wave 9 and only asked in the COVID-19 sub-study if the information was missing.

Other baseline characteristics at Wave 9 that were included in the modelling process was based on those that were shown to have a statistically significant longitudinal association between CVD and quality of life [34]. Age (years) and depression, using the Centre for Epidemiologic Studies Depression Short-Form (CES-D-SF) questionnaire (scored from 0 to 8 where higher scores imply higher levels of depression) were the only continuous variables. The CES-D scale can also be dichotomised to non-severe depression (score < 2) and severe (score ≥ 3) [35]. Categorical variables include body mass index (BMI) which was calculated using height and weight. Weight was collected at Wave 9 but height was collected at Wave 6, which was the latest Wave that height was collected. BMI was categorised, using the England National Health Service(NHS) ranges, into underweight (BMI < 18.5), normal (18.5 ≤ BMI < 25), overweight (25 ≤ BMI < 30), and obese (BMI ≥ 30) [36].

Deprivation was based on a series of nine questions and was scored from one to nine, where 1 signified the least deprived group and 9 signified the most deprived group. The questions pertaining to deprivation were whether or not participants could: (Q1) buy your first choice of food items; (Q2) have family and friends round for a drink or meal; (Q3) have an outfit to wear for social or family occasions; (Q4) keep your home in a reasonable state of decoration; (Q5) replace or repair broken electrical goods; (Q6) pay for fares or other transport costs to get to and from places you want to go; (Q7) buy presents for friends of family once a year; (Q8) take the sort of holidays you want; and (Q9) treat yourself from time to time.

Highest education qualification was grouped into no qualification, foreign/other, NVQ1/CSE, NVQ2/O-level, NVQ3/A-level, higher education below degree, and NVQ4-5/degree level. Participants were asked to describe other members of their households (up to 16 other household members in ELSA Wave 9). If the participant reported zero other people in their household, they were categorised as ‘living alone’. Participants with at least one other person living with them were categorised as ‘not living alone’. Physical activity was grouped into never, 1–3 times a month, once a week, or more than one a week for three levels of activity: mild, moderate, and vigorous intensity. Sex was grouped into males vs. females. Smoking status was divided into never smoked, ex-smoker, and current smoker.

### Statistical analysis

Participants who completed at least one of the COVID Waves were included in the analysis. Baseline characteristics were presented as either mean and standard deviation (SD) or median and inter-quartile range (IQR), depending on if the variable was normally distributed or not.

There are four primary models, two for the CVD outcomes and two for QoL outcomes. The main outcomes were analysed with either a multivariate logistic regression model (newly-developed CVD outcome) or a multivariate linear regression model (change in QoL outcome). All of the previously mentioned baseline characteristics at ELSA Wave 9 were included in the four models. As ethnicity was the main variable of interest, they were added straight at the multivariate model stage of the analysis. Furthermore, newly-developed CVD was used as a predictor for QoL at the same Wave, for example newly-developed CVD at COVID Wave 1 was used as a predictor of QoL at COVID Wave 1.

The association between each baseline characteristic and the outcomes were tested using univariate analyses. Those with p-value ≤ 0.10 were added to the respective multivariate models before the models were finalised by including only variables with p-value ≤ 0.05. Analyses were unweighted at first, with weights being used in sensitivity analyses to account for participants who have answered the questionnaires at all timepoints. Results of weighted analyses found no statistically significant difference between that and of unweighted analyses.

Assumptions of the logistic and linear regression models were tested. For both models, the assumptions that were tested were no multicollinearity among the explanatory variables using the variance inflation factor (VIF), the absence of extreme outliers which were identified using Cook’s distance. Any outliers, defined as any point whose Cook’s distance is more than three times the mean of all distances, would be removed from sensitivity analyses to see if they had a significant influence of the results. Additionally, one assumption of linear models is normality of the residuals, which was tested visually using histograms, and statistically using the Shapiro-Wilks test.

All statistical analyses were conducted using StataSE 17 (64-bit) [37].

## Results

### Baseline characteristics

There were 7,200 participants included in Wave 9 of ELSA, 7,040 participants in Wave 1 of the COVID-19 sub-study, and 6,794 participants in Wave 2 of the sub-study. For the primary analyses, there were 7,362 participant who completed either of the COVID sub-study waves. Table 1 summaries the characteristics of the primary analysis sample. The vast majority of participants were white. Mean age was 67 years, mean BMI was 28 kg/m^2^, with 42% of participants being overweight and over one-quarter obese. Mean depression score was 1.2 with 16% being classed as having severe depression. There was a somewhat even distribution of educational qualifications at NVQ2 (or equivalent) and above, alongside 14% of participants with no qualifications. Most participants lived with at least one other person (80%). As expected, more participants took part in regular mild physical activity compared to more moderate or vigorous intensity. There were more females in the cohort than males (56.5% vs. 43.5%). Half of the participants were ex-smokers, with 8.6% of participants who were still smoking as of Wave 9.

**Table 1:** Summary of participant baseline characteristics at ELSA Wave 9

	Mean or N	SD or %
**Ethnicity**		
White	7,014	95.3%
Non-white	348	4.7%
**Age**	67.3	10.0
**Body mass index**	27.9	5.9
Underweight	43	0.9%
Normal	1,375	29.3%
Overweight	1,966	41.8%
Obese	1,315	28.0%
**CES-D S-F**		
Overall score	1.2	1.7
Non-severe	6,025	83.7%
Severe	1,175	16.3%
**Deprivation score**	1.7	1.6
**Education**		
None	1,010	14.1%
Foreign/other	513	7.1%
NVQ1/CSE or equivalent	256	3.6%
NVQ2/GCE O-level or equivalent	1,610	22.4%
NVQ3/GCE A-level or equivalent	841	11.7%
Higher education below degree	1,133	15.8%
NVQ4/NVQ5/degree or equivalent	1,823	25.4%
**Living alone**		
No	5778	80.3%
Yes	1422	19.8%
**Physical activity**		
**Mild intensity**		
Hardly ever or never	442	6.3%
One to three times a month	156	2.2%
Once a week	616	8.8%
More than once a week	5804	82.7%
**Moderate intensity**		
Hardly ever or never	1064	15.2%
One to three times a month	406	5.8%
Once a week	900	12.8%
More than once a week	4648	66.2%
**Vigorous intensity**		
Hardly ever or never	4096	58.4%
One to three times a month	529	7.5%
Once a week	679	9.7%
More than once a week	1713	24.4%
**Sex**		
Male	3,190	43.5%
Female	4,135	56.5%
**Smoking**		
Never	2,849	41.1%
Ex-smoker	3,488	50.3%
Current	594	8.6%

Abbreviations: CES-D-SF = Centre for Epidemiologic Studies Depression scale - Short Form; GCE = General Certificate of Education; N = Number; NVQ = National Vocational Qualification; SD = Standard Deviation

### Factors associated with newly-developed CVD

Of the 7,362 participants included in the analysis, 7,200 had completed the CVD questions from ELSA core Wave 9. Of them, 1,535 (21%) had CVD at ELSA Wave 9. Of those that did not have CVD at Wave 9 and completed COVID Wave 1 (5,432 participants), 422 developed CVD at COVID Wave 1. Of the participants who did not have CVD at either Wave 9 or COVID Wave 1 and completed Wave 2 (4,742 participants), 135 (2.85%) developed CVD at Wave 2.

Multivariate logistic regression models were carried out for newly-developed CVD, between ELSA core Wave 9 and the COVID sub-study Wave 1 (henceforth named model 1); and between the COVID sub-study Waves 1 and 2 (model 2). Univariate analyses found that all of the baseline characteristics presented in Table 1 were associated with a having newly-developed CVD at COVID Wave 1, but age, BMI, depression, education, moderate and vigorous physical activity, and smoking status were associated with newly-developed CVD at COVID Wave 2, at a 10% significance level. Results of all univariate analyses are presented in Table 2.

**Table 2:** Results of univariate analyses for all of the primary outcome models

	Change in Newly-developed CVD				Change in Quality of life score			
	Wave 9 to Wave 1		COVID Wave 1 to Wave 2		Wave 9 to Wave 1		COVID Wave 1 to Wave 2	
	χ^2^	P	χ^2^	P	F	P	F	P
**Age**	27.77	< 0.001	15.11	< 0.001	8.42	0.004	0.15	0.697
**BMI**	13.54	0.004	20.88	< 0.001	2.36	0.069	0.89	0.448
**CES-D-SF**	36.20	< 0.001	8.09	0.005	25.65	< 0.001	0.62	0.430
**Deprivation score**	14.63	< 0.001	0.82	0.364	19.55	< 0.001	0.28	0.594
**Highest educational qualification**	22.63	0.001	19.15	0.004	0.92	0.477	1.27	0.269
**Living alone**	13.68	< 0.001	0.91	0.340	55.04	< 0.001	0.05	0.830
**Mild physical activity**	15.95	0.001	3.54	0.316	30.32	< 0.001	1.07	0.359
**Moderate physical activity**	45.37	< 0.001	11.67	0.009	35.16	< 0.001	0.42	0.741
**Vigorous physical activity**	32.98	< 0.001	11.39	0.010	8.40	< 0.001	0.42	0.736
**Sex**	5.35	0.021	0.06	0.801	33.90	< 0.001	0.16	0.694
**Smoking status**	35.02	< 0.001	4.67	0.097	3.70	0.025	0.52	0.596
**Newly-developed CVD**	NA	NA	NA	NA	25.57	< 0.001	0.01	0.925

Abbreviations: BMI = Body Mass Index; CES-D-SF = Centre for Epidemiologic Studies Depression scale - Short Form; COVID = Coronavirus Infectious Disease; CVD = Cardiovascular Disease; NA = Not Applicable

For model 1, the logistic regression analysis suggested that age, depression, deprivation, and regular moderate physical activity were all significant predictors in a participant developing CVD at COVID Wave 1 when they did not have one at ELSA Wave 9. Higher age, depression, and deprivation score were associated with a higher odds of developing CVD, whereas regular moderately and vigorously-intense physical activity was associated a lower odds of developing CVD. Moreover, females had a 34% reduced odds of developing CVD during lockdown compared to males.

Age, BMI, and depression were the only statistically significant predictors in model 2. One-unit increased in age and depression score increased the odds of newly-developed CVD between COVID Waves 1 and 2 by 4% and 13%, respectively. Underweight participants saw the biggest increase in odds of developing CVD, by over 5 times, compared to participants in the normal BMI range. Overweight and obese BMI also increased the odds by 35% and almost 3 times, respectively. Results for both models are presented in Table 3.

Assumptions of the logistic regression models were met. This included acceptable levels of multi-collinearity based on variance inflation factor and pairwise correlations. When re-running the two models without highly-influential observations, there were no statistically significant difference in odds ratios. Finally, both models were statistically significant, model 1 (chi-squared p < 0.001), model 2 (chi-squared p < 0.001).

**Table 3:** Results of the logistic regression analyses with newly-developed CVD as the outcome at two timepoints

	Change from Wave 9 to Wave 1				Change from Wave 1 to Wave 2			
	OR	LCI	UCI	P	OR	LCI	UCI	P
**Constant**	0.03	0.01	0.10	< 0.001	0.001	0.0002	0.01	< 0.001
**Age**	1.02	1.01	1.04	0.010	1.04	1.01	1.06	0.002
**BMI**								
Normal (reference)					0.00			
Underweight					5.46	1.50	19.91	0.010
Overweight					1.35	0.77	2.37	0.301
Obese					2.77	1.59	4.81	< 0.001
**CES-D-SF**	1.12	1.03	1.21	0.007	1.13	1.02	1.26	0.018
**Deprivation score**	1.14	1.04	1.25	0.007				
**Moderate physical activity**								
Hardly ever or never (reference)	0.00							
One to three times a month	0.30	0.13	0.73	0.008				
Once a week	0.68	0.38	1.20	0.179				
More than once a week	0.68	0.45	1.04	0.072				
**Vigorous physical activity**								
Hardly ever or never (reference)	0.00							
One to three times a month	0.79	0.41	1.54	0.492				
Once a week	0.56	0.28	1.11	0.095				
More than once a week	0.50	0.30	0.84	0.009				
**Sex**								
Male (reference)	0.00							
Female	0.66	0.47	0.91	0.012				

Abbreviations: BMI = Body Mass Index; CES-D-SF = Centre for Epidemiologic Studies Depression scale - Short Form; LCI = Lower 95% Confidence Interval; NVQ = National Vocational Qualification; OR = Odds Ratio; UCI = Upper 95% Confidence Interval

### Factors associated with quality of life

A total of 7,362 participants completed the CASP-19 questionnaire at ELSA Wave 9. As the score for each individual question was available, only the 12 questions that are included in the CASP-12 score was summed to acquire the CASP-12 score at Wave 9 (mean = 26.8; SD = 5.89) and allow direct comparability between all waves in this analysis. At COVID Wave 1, 7,040 participants completed CASP-12 with a mean score of 26.0 (SD = 6.23). At COVID Wave 2, 6,794 participants completed CASP-12 with a mean score of 25.51 (SD = 6.39). The mean change in CASP-12 score from Wave 9 to COVID Wave 1 was − 0.78 (SD = 6.19), and from COVID Waves 1 to 2 was − 0.60 (SD = 4.04).

Multivariate linear regression models were used to model the change in QoL score between ELSA Wave 9 and COVID Wave 1 (model 3), and COVID Waves 1 and 2 (model 4). Univariate analyses found that all of the baseline characteristics except for highest educational qualification and smoking status were associated with change in QoL from ELSA Wave 9 to COVID Wave 1. None of the baseline characteristics were significantly associated with change in QoL from COVID Wave 1 to Wave 2.

The analysis for model 3 concluded that ethnicity, depression score, both mildly and moderately-intensive physical activity, gender, and newly-develop CVD were statistically significant predictors of change on QoL. Non-whites, higher depression, female gender, and having a newly-developed CVD condition were associated with a decrease in QoL score from.

A one-score increase in depression (signifying higher levels of depression) was associated with a 0.24 reduction in overall CASP-12 score. More regular physical activity increased QoL score from COVID Wave 1 to Wave 2, and females were associated with a reduction in QoL of 1.30 compared to males. Whereas increases in age and more regular mildly or moderately-intensive physical activity were protective against decreased QoL score during lockdown.

Model 4 contained no variables as p > 0.05 for all of the variables, including ethnicity (p > 0.33).

Results of model 3 are presented in Table 4.

As with the logistic models, assumptions of the linear regression model were also met, including acceptable levels of multi-collinearity based on variance inflation factor and pairwise correlations. When re-running the model 3 without highly-influential observations, there were no statistically significant difference in odds ratios. The regression model was also statistically significant (p < 0.001). The adjusted R^2^ indicated that 3% of the variance of the change in QoL could be explained by the model.

**Table 4:** Results of the logistic regression analyses with change in QoL as the outcome at the first timepoint only

	Change in QoL from Wave 9 to Wave 1			
	Estimate	LCI	UCI	P
**Constant**	-3.48	-4.84	-2.13	< 0.001
**Ethnicity**				
White (reference)	0.00			
Non-white	-0.81	-1.60	-0.01	0.046
**Age**	0.03	0.01	0.04	< 0.001
**CES-D-SF**	-0.24	-0.34	-0.15	< 0.001
**Mild physical activity**				
Hardly ever or never (reference)	0.00			
One to three times a month	1.37	0.14	2.60	0.029
Once a week	1.19	0.32	2.05	0.007
More than once a week	1.65	0.92	2.38	< 0.001
**Moderate physical activity**				
Hardly ever or never (reference)	0.00			
One to three times a month	0.83	0.05	1.61	0.038
Once a week	0.76	0.13	1.38	0.018
More than once a week	0.88	0.37	1.39	0.001
**Sex**				
Male (reference)	0.00			
Female	-0.80	-1.11	-0.48	< 0.001
**Newly-developed CVD at Wave 1**				
No (reference)	0.00			
Yes	-1.03	-1.60	-0.45	< 0.001

Abbreviations: BMI = Body Mass Index; CES-D-SF = Centre for Epidemiologic Studies Depression scale - Short Form; LCI = Lower 95% Confidence Interval; NVQ = National Vocational Qualification; UCI = Upper 95% Confidence Interval

## Discussion

### Main findings

To the authors’ knowledge, this is the first study to investigate the factors associated with developing CVD in adults aged 50 years or older living during two periods of the 2020 COVID-19 lockdown in England, and factors which predicted an increase or decrease in quality of life during the same timepoint in the same population. The results presented in this paper are novel as it explores the dynamic between the already-known factors associated with CVD and QOL during an unprecedented time in recent years. The present study found that higher age, higher depression score, higher deprivation score, reduced physical activity, and being male significantly increased the odds of developing CVD during the first lockdown. However, only increased age, being underweight or obese significantly increased the odds of developing CVD during the second phase of lockdown later in 2020. The factors associated with QOL followed a similar story. Many factors were identified that were significantly associated with a change in QOL during the first lockdown but were non-significant in the second wave of the COVID-19 sub study. This included the Non-white ethnicity, higher age, higher depression score, lower levels of physical activity, being female and newly-developed CVD are associated with a decrease in quality of life during the coronavirus pandemic. In our study, we found that the first lockdown period was a critical time for addressing risk factors for developing CVD and for maintaining quality of life (QOL), particularly for certain vulnerable groups. This is in line with previous research that has highlighted the importance of focusing on modifiable risk factors such as physical activity, body mass index (BMI), and mental health during periods of major societal disruption, such as a pandemic lockdown [38]. During such periods, physical activity levels can decrease and BMI can increase, exacerbating the risk of CVD. Mental health issues, such as depression, can also become more prevalent and further impact on QOL [39].

Ethnicity was not associated with developing CVD during the 2020 lockdown in the UK when a participant did not have CVD prior to lockdown, but it was a statistically significant predictor of QoL throughout lockdown, where Non-whites had lower levels of QoL compared to their White counterparts. A systematic literature review by Patel [40] found that the majority of non-white ethnicities suffered from worse mortality outcomes when suffering from CVD, which may worsen QoL. Newly-developed CVD was associated with QoL only during the first wave of the ELSA COVID-19 sub-study, but not for the second wave. Those with new CVD had lower levels of QoL, and this corresponds to results found in a study by Patel et al [34], where CVD was associated with QoL in the first couple of waves post-baseline. An explanation of this might be that those with CVD may struggle to manage their CVD straight away, but through lifestyle changes and treatment, they may feel better and feel their QoL has increased over the coming months or years.

As expected, age was associated with both CVD and QoL. With a mean age of this cohort being 67 years, increases in age led to poorer QoL and increased odds of developing CVD. Age is an independent risk factor for CVD in adults over 50 years [41], and QoL starts off poorer and declines faster for older individuals [42], which was seen in the present study.

Depression score, using the CES-D-SF questionnaire, was associated with both CVD and QoL. When modelling CVD, a one-score increase in depression score was associated with a 12% and 13% increased odds of new CVD at COVID Waves 1 and 2, respectively. Potential mechanisms that link depression with an increased CVD risk include poor adherence to multiple risk reducing behaviours including reduced physical activity, increased smoking, and decreased adherence to cardiovascular medicines [43–45]. This has a knock-on effect with QoL of those with CVD as depression is highly prevalent in patients with CVD [46] which, in turn, affects QoL. Among cardiovascular patients, depression, and anxiety, were found to have full mediating effects on life satisfaction and QoL [47].

Sex was statistically significantly associated with both newly-developed CVD and QoL at COVID Wave 1. Females had a reduced odds of developing CVD compared to males but, conversely, they also had on average a lower QoL compared to males. The results of the present study coincides with a study by Bucciarelli and colleagues [48] where they postulated that, as primary caregivers within their own families, women had an increased familial and social burden placed on them during the lockdown [49], such as home schooling and childcare, leading to increased stress and reduced QoL.

Regular physical activity, at mild to vigorous intensity, was also a predictor of new CVD and QoL, where those who exercise more regularly were associated with lower odds of developing CVD, and higher QoL, similar to previous studies [50, 51]. During the first lockdown in March 2020 and subsequent strict quarantine rules, it is feasible that the ageing population were unable to even take a regular walk outside of the house. This can possibly cause even more harmful effects on older people who are more likely to be living alone or in care homes away from family. Coupled with the deleterious impact that COVID-19 has had on cardiovascular risk factors such as unhealthy food habits, and delayed critical care due to the fear of the contagion [52], this would naturally cause decreases in healthy lifestyle choices and lead to poorer outcomes.

### Strengths and limitations

This is the first paper to look at the interplay between CVD and QoL and how they changed during beginning of the coronavirus pandemic and the first two lockdowns in the UK. The uniqueness of the current study is that it is an extension of an earlier paper [34], which examined the association between key baseline characteristics, CVD, and QoL over the nine core waves of ELSA, but now through the eyes of a global pandemic. It investigated how one of the most at-risk groups, the ageing population of people aged 50 years and above, were affected by the pandemic, and how the factors related to QoL differed pre and post-pandemic.

The longitudinal nature of this study is a key strength as it allows changes in CVD and QoL over time, not just at one static timepoint like in cross-sectional studies. This allows temporal changes in the outcomes to be measured and see what factors correlate to the trends of these outcomes. ELSA is an observational study so causation cannot be assumed, only correlation. Furthermore, despite the longitudinal design of this paper, only three timepoints were analysed, with the latter two only a few months apart from each other. Future work assessing multiple timepoints over more consistent periods would be beneficial. The study analysed validated questionnaires using robust statistical analysis, along with comprehensive post-estimation and assumption testing. This study relied on self-reported data, which introduces potential recall bias, and the amount of missing data for key variables.

Additionally, White participants made up the vast majority (95%) of the ELSA study during the waves analysed in this paper. As ethnicity is a major factor in CVD, it may have played a significant role in the models analysed, but due to the difference between the number of White and Non-white participants, this effect may have been missed out.

The ELSA project is a survey of people in England living in residential homes only. This could pose a generalisability issue as it misses out the experiences of people living in care or nursing homes, or in hospital., however small in quantity they may be These people may be most at-risk than the general population, so an estimate for these people would be valuable also.

### Implications and future research

The results of this paper are important as knowing the demographics which are the most at-risk allows limited health services and resources to be directed to these key groups and allocated much more effectively. Due to the lockdown, women had increased responsibilities in the household which affected their QoL. This was also the case with people unable to exercise or partake in physical activity while being stuck at home. Furthermore, quarantines increased depression, possibly due to loneliness, in people and lowered QoL.

Future work assessing the longitudinal trend of QoL over more timepoints that are consistently spaced out will be beneficial to see long-term effects of CVD on QoL, and if that echoes results found elsewhere where CVD affects QoL only in the short-term, and how this is affected by a worldwide pandemic. Interactions between women and how their workload changed during lockdown, and lack of physical activity or socialising should be studied also.

## Conclusion

This study identified the risk factors that predicted developing CVD and changes in QoL in adults over 50 years during the COVID-19 lockdown in the UK in early and late 2020. Risk factors of both outcomes included age, depression, deprivation index, gender, and physical activity. CVD was a predictor of low QoL only at the first COVID wave, not the second. Ethnicity was associated with QoL, where Non-white participants had lower QoL compared to Whites. In conclusion, the results of this study are significant as they show how the coronavirus pandemic and subsequent lockdown orders influenced developing CVD and how that played a part in a decrease in QOL for participants of ELSA.

## Data Availability

The datasets generated and/or analysed during the current study are available in the UK Data Service (https://beta.ukdataservice.ac.uk/datacatalogue/series/series?id=200011).
